# Effect of radiant catalytic ionization on environmental conditions in rodent rooms and the haematological status of mice

**DOI:** 10.1186/s12917-022-03402-5

**Published:** 2022-08-03

**Authors:** Tomasz Niemiec, Krzysztof Skowron, Wiesław Świderek, Joanna Kwiecińska-Piróg, Grzegorz Gryń, Urszula Wójcik-Trechcińska, Marta Gajewska, Klara Zglińska, Andrzej Łozicki, Piotr Koczoń

**Affiliations:** 1 Institute of Animal Sciences, Warsaw University of Animal Sciences, Warsaw, Poland; 2 Department of Microbiology, Nicolaus Copernicus University in Torun, Ludwik Rydygier Collegium Medicum, Bydgoszcz, Poland; 3 Plant Breeding and Acclimatization Institute – National Research Institute, Bydgoszcz, Poland; 4 Department of Genetics, Maria Sklodowska-Curie National Research Institute of Oncology, Warsaw, Poland; 5 Institute of Food Sciences, Warsaw University of Life Sciences, Warsaw, Poland

**Keywords:** Radiant catalytic ionization, Laboratory animal, Microorganisms, Ammonia, Dust, Health status

## Abstract

High stocking densities, closed animal houses, and elevated concentrations of bacteria, fungi, and the products of their activity, including ammonia and hydrogen sulphide, have adverse health effects. Active techniques used to reduce unfavourable environmental conditions, such as ventilation, sprinkling, bedding sorbents, and nutritional treatments, are not always sufficient to improve the animals’ living environment. The current paper aims to evaluate the effect of radiant catalytic ionization (RCI) on airborne microorganisms, cage microbiological status, gaseous ammonia concentrations, and the haematological status of mice in animal houses. After one week of operation of an RCI system, the number of airborne bacteria and fungi in the experimental room decreased in comparison to the first day of the experiment (*p* < 0.05 and *p* < 0.05 respectively), as did the concentrations of ammonia (*p* < 0.01) and dust. At the same time, the basic health parameters of the mice, determined in the blood, were very similar between the control and experimental room. RCI seems to be an ideal solution to ensure high hygiene standards in animal rooms and houses with limited use of disinfectants or antibiotic treatment of sick animals. An additional, environmental benefit is the limited amount of nitrogen released.

## Introduction

Airborne microbial contamination is one of the most serious problems affecting indoor air, posing a significant threat to the health of laboratory animals. Several air-cleaning techniques are employed to remove microorganisms. These include: independent ventilation ducts, micro-isolator animal cages and individually ventilated cage (IVC) systems equipped with HEPA filters that eliminate most pathogens from the air of the room or cage [1–3]. Another method is to combine HEPA filters with photocatalytic systems to degrade microorganisms on the surfaces of the filters [4]. The vaporized hydrogen peroxide (VHP) sterilization technique and UVC lamps, widely used for disinfecting laboratory rooms, are effective preventive methods. However, they can only be used periodically and when staff and animals are not present [4, 5], An exception is lamps emitting wavelengths of 222 nm, as research demonstrates that KrCl lamps can efficiently inactivate pathogens without inducing acute reactions in the skin or eyes or delayed effects such as skin cancer [6]. Improving hygiene or sanitation on breeding farms takes place through the use of numerous chemicals, including water solutions of hydrogen peroxide [7, 8]. However, several studies reports raise concerns about the possibility of inadequate use of H_2_O_2_ and increasing microbial resistance to common disinfectants [9].

Most research on chemical and physical hazards in animal housing is carried out on livestock. Particles in livestock houses are believed to adversely affect the respiratory health of both animals and humans [10–12]. Elevated PM10 (particulate matter with an aerodynamic diameter ≤ 10 μm) concentrations can increase the risk of chronic bronchitis, asthma-like symptoms, cardiovascular disease, pneumonia lesions, and lung cancer among farmers and livestock [13, 14]. Farmers use numerous technological solutions to reduce dust emission in animal houses. The most commonly used systems are fixed oil spraying systems, negative or positive air ionization, dry filters, electrostatic precipitators, and manure drying tunnels that efficiently reduce airborne particulate matter (PM) [15]. Another major contributor to air pollution in livestock houses is ammonia (NH_3_), which can be generated from decomposition of faeces and urine by microorganisms. Particles (PM) can adsorb ammonia and carry it for a long time [16, 17]. Ammonia in housing facilities can adversely affect animals' performance, welfare [18], feed intake, and growth rate [19]. Various hygienic methods are used to reduce emissions of ammonia and other odorous gases in livestock facilities. Ammonia emissions are reduced by adding chemical, mineral, or microbiological additives to the bedding, which reduces ammonia levels and has a bactericidal and deodorizing effect [20, 21]. The most commonly used additives are formaldehyde, quicklime, superphosphate, organic acids (acetic or propionic), and various fungistatic preparations. An alternative to chemical compounds are natural additives such as zeolites, dolomites, certain types of brown coal, peat preparations, or those containing saponins [22–24]. The environmental balance in animal rooms can be ensured by a system that operates in a continuous cycle and actively (not passively) reduces undesired airborne and surface-borne microorganisms. One technology based on photocatalysis that can be used in the presence of people and animals is radiant catalytic ionization (RCI). Devices based on RCI technology are intended for installation in heating, ventilation, and air conditioning systems or for use in stand-alone form, independent of existing systems [25]. They emit hydrogen peroxide in trace concentrations (< 0.0328 ppm) safe for humans and animals [26]. In 2020, the Food and Drug Administration (FDA) approved RCI technology (ActivePure) deployed in an Areus Medical Guardian unit as a Class II Medical Device for use in occupied rooms to inactivate six different pathogens, including an RNA virus similar to SARS-CoV-2 [27]. In addition to low-levels of hydrogen peroxide, RCI releases negative ions. The combination of hydrogen peroxide and negative ions generated during radiant catalytic ionization effectively inactivates gram-positive and gram-negative bacteria, fungi, and viruses. The hydrogen peroxide damages various cellular sites, destroying proteins and genetic material, inactivating cellular enzymes, and disturbing metabolic pathways [28–30]**.** Another advantage of photocatalytic air purification is its ability to fully mineralize volatile organic compounds (VOCs) to CO_2_ and H_2_O (Fei et al. 2021). Kowalski et al. [3]reported that titanium dioxide and UV irradiation selectively oxidize ammonia, with nitrogen and water as the main reaction products [31]. In addition to reducing levels of microorganisms, photocatalysis can reduce the products of their metabolism, including ammonia.

The concentration of H_2_O_2_ emitted by devices based on RCI technology is 10 times higher (0.03 ppm) than that present in the outside air in summer [32], but 10 times lower (0.3 ppm) than the permissible concentrations specified in European Union regulations. Test results show that the NOAEL (no observed adverse effect level) for symptoms of pulmonary dysfunction is in the range of 0.1–0.6 ppm. Irritating effects in humans have been observed after exposure to the compound at a concentration of 0.6 ppm, which has been recognized as the LOAEL value (lowest observed adverse effect level). This was used to calculate the permissible exposure limit (PEL) for hydrogen peroxide, which is 0.4 mg/m^3^. Vapour from high concentrations of hydrogen peroxide is irritating to the respiratory tract, skin, and eyes. Hydrogen peroxide in exposed humans has been shown to irritate the skin (20 mg/m^3^, 4 h), mucous membranes of the respiratory tract, and eyes (LOAEL 10 mg/m^3^), while at concentrations of 5 mg/m^3^ (NOAEL) or lower it did not cause symptoms of irritation in the mucous membranes of the respiratory tract or eyes [33].

Although devices using RCI technology have proven useful in public utility buildings, they require further scientific investigation for widespread global acceptance with regard to their effectiveness and the safety of laboratory animals and livestock. Therefore, the research question posed in the current study is whether RCI technology in rodent housing improves its environmental status without affecting the basic haematological parameters of mice.

## Materials and methods

### Animals and housing

The study was carried out using 320 two-month-old mice (160 males and 160 females) from an outbred herd of L and C selection lines derived from inbred A/St, BALB/c, and BN/a C57BL/6 J strains from the rodent house of the Institute of Animal Sciences, Warsaw University of Life Sciences. The experiment preceded the herd selection stage as part of ongoing breeding procedures. The mice were divided into two groups and placed in two identical rooms (58.8 m^3^) at 22 ± 2˚C, 55 ± 10% humidity, and a 12/12 h light/dark cycle. The animals had free access to water and complete feed (Labofeed H, Morawski, Poland). Softwood bedding granules (TierWohl Super) were sterilized using UVC and added to each cage. For environmental enrichment, paper tubes were placed in each cage. The study was conducted at a conventional facility with standard weekly cage and room cleaning procedures (including UVC sterilization). The animals undergo microbiological, parasitological and virological tests once a year. The pressure of all animal rooms was positive with respect to the hallways. Rooms were ventilated using 18 full air exchange cycles of 1,100 m^3^ of fresh air per hour. The intake duct of the ventilation system was equipped with standard F8 fine filters (EN 779:2002). Both groups/rooms were standardized with respect to environmental conditions (temperature and humidity) and the line and sex of animals. Each room contained three stainless steel racks holding 40 conventional plastic cages (Tecniplast, Milan, Italy) with dimensions of 31 × 16x14 cm. There were four animals (male or female) in each cage, with a floor area of 496 cm^2^. The average weight of the mice in the control group and the RCI group was 11.58 g ± 2.3 SD and 11.26 g ± 1.73 SD, respectively. After seven days of the experiment, 20 animals (five males and five females per group/room) were euthanized in a separate room dedicated for this purpose, as part of routine culling of animals from the breeding herd. Blood samples were collected to analyse the health status of the animals. The experiment did not include any procedures that required the approval of the Ethics Committee for Animal Experimentation (statement issued by the Second Local Ethics Committee for Animal Experimentation on 16 June 2021). One day before the start of the experiment, routine maintenance (bedding replacement and wall and floor washing) was carried out in the rooms. During the seven days of the experiment, laboratory staff came into the room once a day to replace the water and food and perform animal health checks.

### Device with RCI technology (AP 3000): description and function

**Fig. 1:**
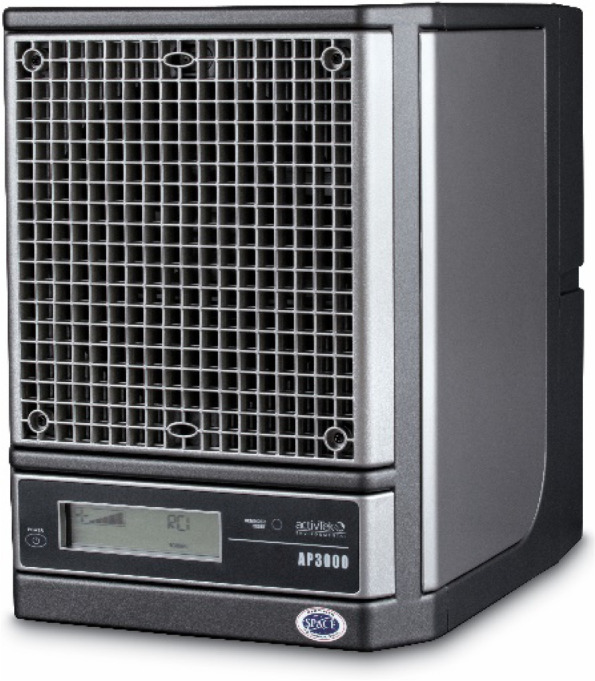
AP 300 radiant catalytic ionization device

**Fig. 2:**
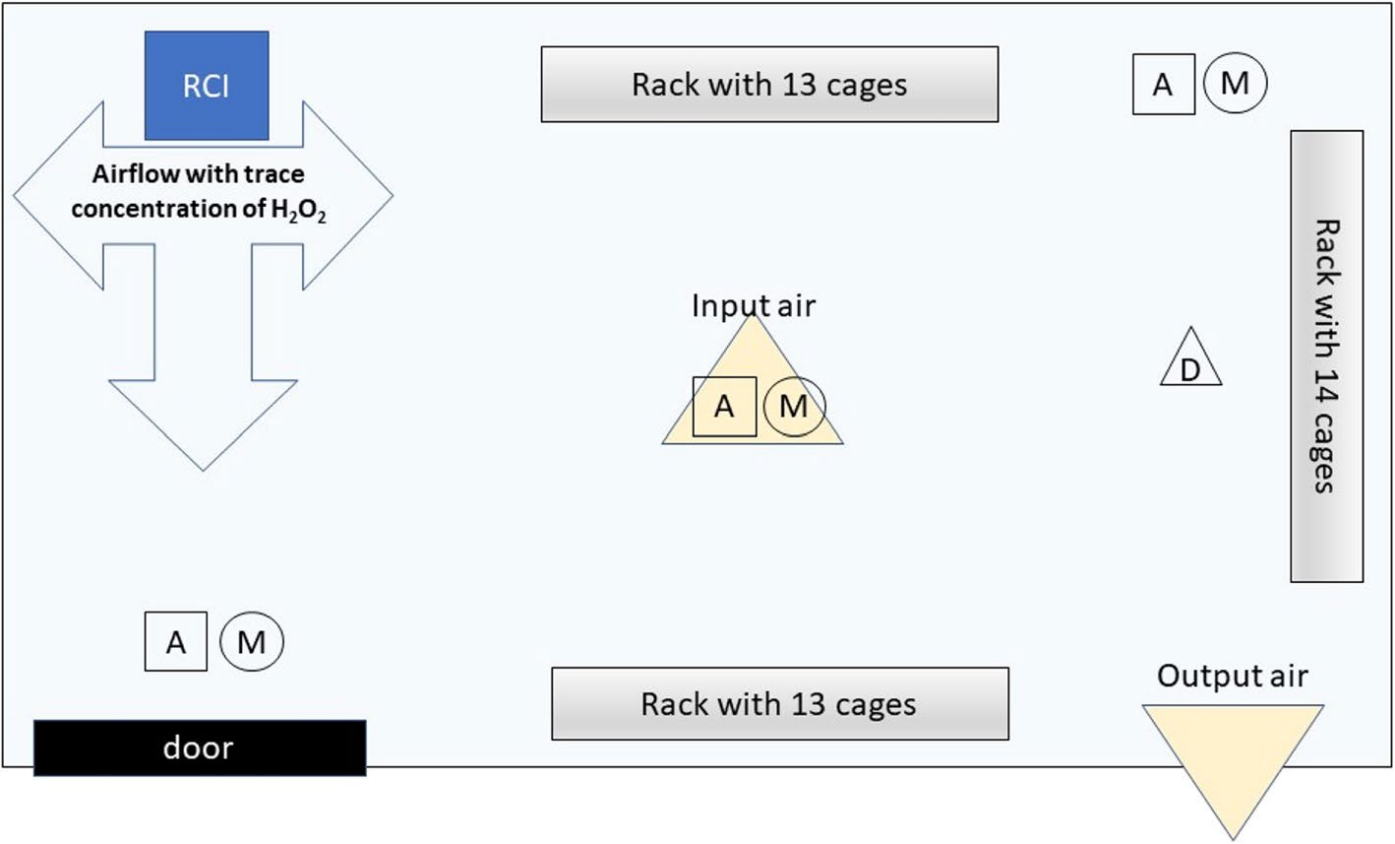
Diagram of the RCI room with the location of all elements, including the RCI instrument, airflow (input and output) and sampling sites (M – microbiological, A – ammonia, D – dust)

The air flow of the AP 3000 (ActivTek Sp. z o. o., Kielce, Poland) (Fig. 1) was driven by a fan (220 v, 50/60 Hz, 50 W) with a diameter of 120 mm. The maximum air capacity of the device was 233 m^3^ h^−1^. The RCI device consisted of a matrix of elongated tubular components made of polycarbonate, arranged in parallel in a honeycomb-like pattern. The RCI device was equipped with two matrices consisting of 406 tubular components each. Each tube had a diameter of 4 mm and a length of 15 mm. The total active surface area of both matrices was 763·28 cm^2^. The coating of the basic elements of the matrix had hydrophilic properties and contained titanium dioxide. Opposite the system was an 8 W UV lamp, which was the source of ultraviolet radiation. The UV lamp generated UV rays with wavelengths of 185 and 254 nm. As a result of the catalytic oxidation stimulated by UV radiation, reactive oxygen species were generated at the boundary of heterogeneous phases (gas–solid). The total number of ions generated was ~ 5 × 10^4^ per cm^−3^ of air. Detailed information on RCI devices and their technology is available in patent no. US 8,585,979 B2. The RCI cells produced low levels of hydrogen peroxide (H_2_O_2_) gas and superoxides (O^2^-). ROS generation and antibacterial mechanism of action of the device with RCI technology were described by Skowron et al. [34]**.** The AP 3000 device was located in the experimental room, one metre above the floor, facing the entrance door (Fig. 2). The unit was permanently switched on during the seven days of the experiment.

On the first and seventh days of the experiment, air and surface samples were collected to determine the total number of microorganisms and the levels of ammonia and dust. The first samples were taken before the RCI device was turned on. At the end of the experiment, the samples were taken immediately after the RCI was turned off. Settle plates were used for qualitative analysis of airborne microorganisms. The media plates were placed on the room's diagonal line (endpoints and centre) at a height of 1 m from the floor and exposed for 30 min. Samples were taken at 8 a.m., before the start of routine animal handling, and then incubated. Three cages were examined in each room. One cage was selected from the centre of each rack in both rooms. Five samples of approximately equal size were collected from each cage from the surface layer of the bedding (up to 1 cm deep), both soiled with urine and faeces and unsoiled, from which an aggregate sample weighing 100 g was made. Samples were placed in sterile containers and delivered to the laboratory within four hours. The air was collected from three points with two repetitions for ammonia and from one point for dust in each of the two rooms. Dust particles were collected using a PCXR4 air sampling pump (SKC, USA). Ammonia samples were collected using a Low Flow Sampler, LFS 113D (Gilian, USA). On the completion of the experiment, after seven days, deep anaesthesia was induced in the animals (by isoflurane), followed by cervical dislocation by trained and experienced staff. Blood samples from the heart were collected in heparinized tubes, cooled to 4 °C, and submitted for analysis. The animals were euthanized as part of routine culling.

#### Microbiological approach

##### Air

Three different media were used to grow a wide spectrum of microorganisms: Columbia 5% Sheep Blood Agar (CAB, Becton Dickinson), Tryptic Soy Agar (TSA) (BIOMÉRIEUX), and Sabouraud Agar (SAB, Becton Dickinson). A microbiological assessment of the air in the control and experimental rooms was performed twice (before and after the experiment), and was based on three replicates of each medium at three measurement points. The TSA plates were incubated for 72 h at 37° C and a further 14 days at 28 °C. Based on the plate analysis of the number of colony-forming units (CFU/m^3^), the number of microorganisms in 1 m^3^ of air was determined.

##### Cage

Samples of bedding were transferred to 500 ml of sterile phosphate-buffered saline solution (PBS, BTL) and shacked for 60 min to release the microbes in the solid components of the bedding into the solution. Next, serial dilutions (10^0 – 10^-6) of the solution were made using sterile PBS. Two independent series were prepared for each sample. A 100 µl sample of each dilution was cultivated on Columbia 5% Sheep Blood Agar (CAB, Becton Dickinson) and Sabouraud agar (SAB, Becton Dickinson). CAB plates were incubated for 48 h at 37 °C and then a further 48 h at room temperature. SAB plates were placed at 37 °C for 24 h and transferred to room temperature for the next four days. The resulting colonies were counted using a colony reader (Galaxy 330). Results were expressed as the number of colonies in 1 g of bedding.

#### Ammonia and dust levels in the air

The level of gaseous ammonia was determined according to Polish regulation PN-71/Z-04041 [35] using Nessler’s reagent colorimetric method. The samples were scanned using a DR 4000 UV–VIS spectrophotometer (Hach, USA). Organic dust of animal and plant origin, excluding wood and flour dust, was analysed according to regulation PN-91/Z-04030/05 [36] by the gravimetric method. The flow rate for collecting dust was calibrated to 2.0 L min^−1^ for total dust.

#### Haematological status parameters

Red blood cell (RBC) count, haematocrit, mean cell volume (MCV), haemoglobin concentration, and white blood cell (WBC), neutrophil, lymphocyte, and platelet counts were determined using standard methods with a CELL-DYN 3700 analyser (GMI, USA).

### Statistical analyses

As the microbiological results were not of a normal distribution, non-parametric median tests were used for statistical analysis. Medians from different experimental groups were compared using the Mann–Whitney U test and Kruskal–Wallis test (Statistica software package). The health status results were analysed using parametric tests (ANOVA and Duncan’s range test) in StatGraphics 4.1 Plus (StatPoint, Inc., USA).

## Results

### Microbiology of the environment

**Fig. 3:**
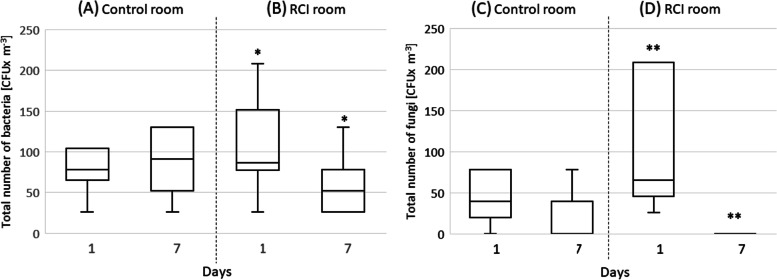
CFU differentiation for total airborne bacteria and fungi between the control room and RCI room on the 1st and 7th days of the experiment, significantly different at *p < 0.05 and **p < 0.05, Kruskal–Wallis test

*Based on the phenotypic identification, the following microorganisms were identified, Basa-D (Micrococcus spp., Staphylococcus spp., pseudomycelia, gram-negative cocci), TSA (Micrococcus spp., Staphylococcus spp., Streptococcus spp., Proteus mirabilis, gram-negative coccobacilli), Chapman Agar (Micrococcus spp., Staphylococcus epidermidis, Staphylococcus xylosus, Bacillus spp., pseudomycelia), Sabouraud agar (hyphomycetes and yeast-like fungi).* The numbers of airborne bacteria and fungi found in the rooms (control and RCI) during the experiment are presented in Fig. 3. In the room with the RCI device, after seven days of the experiment, there was a statistically significant reduction in the number of bacteria (*p* < 0.05) and fungi (*p* < 0.05) in the air, compared to the first day of an experiment. The numbers of airborne bacteria and fungi in the control room did not change during the seven days of the experiment. The difference in the numbers of bacteria and fungi in the air between the control room and the RCI room on the first day of the experiment was not statistically significant.

**Fig. 4:**
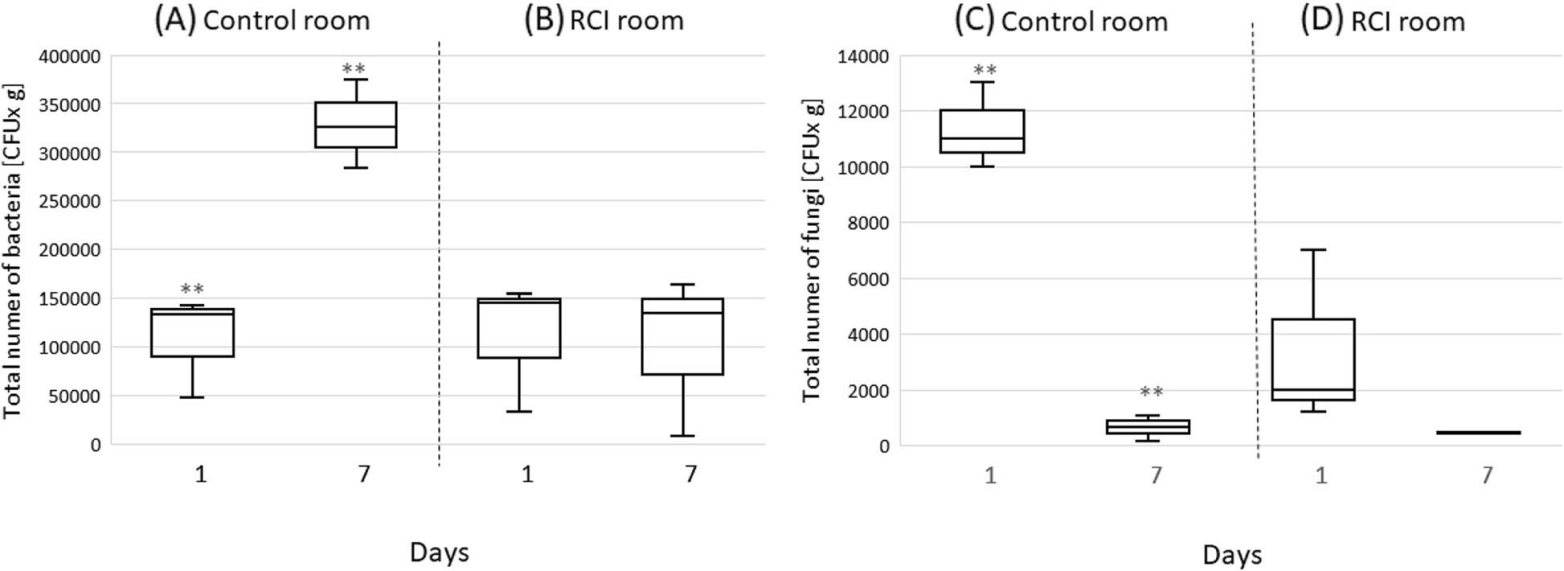
Bacterial and fungal CFUs in the bedding of cages in the control and RCI room on the 1st and 7th days of the experiment, **significant difference at *p* < 0.05, Kruskal–Wallis test

The bedding in the cages of the control room showed a significantly (*p* < 0.05) higher number of bacteria (see Fig. 4A) but a significantly (*p* < 0.05) lower number of fungi on the seventh day of the experiment than on the first day (Fig. 4C). In the RCI room, there were no differences between the number of bacteria (Fig. 4B) on the seventh day and the first day of the experiment, while the number of fungi decreased after seven days of RCI activity, but not significantly (Fig. 4D).

### Ammonia and dust in the air

**Fig. 5:**
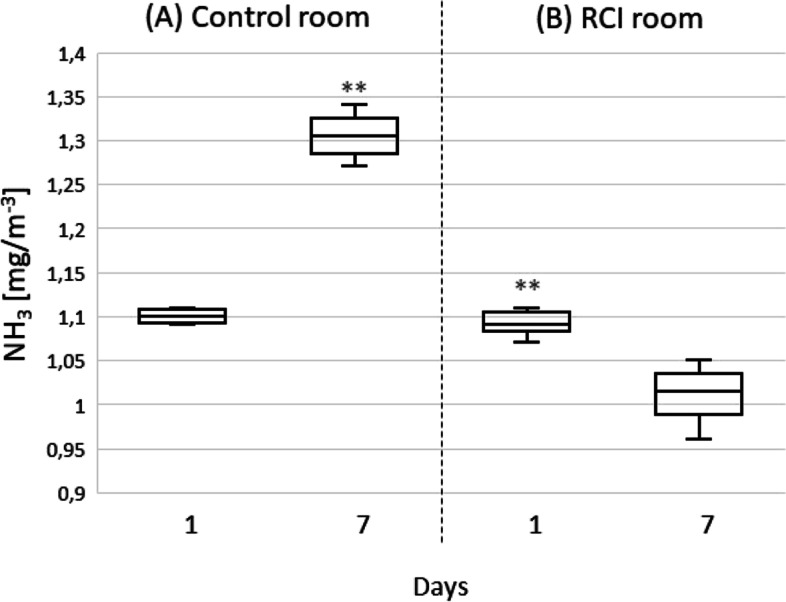
Effect of 7 days of RCI on ammonia gas concentrations in mouse rooms, * significantly different at **p < 0.01, Mann–Whitney U test

The ammonia gas concentration in the control room on the seventh day was significantly higher (*p* < 0.01) than on the first day of the experiment. In the RCI room, the ammonia concentration was reduced over the seven days of RCI treatment (Fig. 5). After seven days of RCI activity, the dust concentration remained stable, while the dust in the control room increased (Table 1).

**Table 1:** Dust concentrations in the control and RCI rooms

Group	Control room		RCI room	
Day	1	7	1	7
Dust [mg/m^3^]	0.13	0.53	0.13	0.14

### Haematological status of mice

The haematological parameters (Table 2) of the mice were very similar between the control and the RCI groups, indicating that RCI had no effect on these parameters.

**Table 2:** Haematological parameters in peripheral blood of control and RCI mice

Parameter	Reference values^a^	Control room	RCI room	SEM	*P*-value
RBC^b^ (T/l)	7.1–9.5	9.81	9.88	0.1474	0.7372
Haematocrit (%)	37.4–51.7	48.65	48.70	0.2746	0.9038
MCV^b^ (fl)	41–57.4	49.6	49.25	1.0585	0.8266
Haemoglobin (mmol/l)	7.19–9.81	9.61	9.70	0.0408	0.1756
WBC^b^ (G/l)	1.5–4.8	3.45	3.21	0.6921	0.8147
Neutrophils (%)	11–29	20.5	18.3	3.4200	0.2760
Eosinophils (%)	0–5	0.55	0.47	0.0790	0.5338
Basophils (%)	0–1	0.62	0.67	0.1674	0.8492
Monocytes (%)	0–6	1.15	1.72	0.2306	0.1612
Lymphocytes (%)	65–87	77.23	78.82	2.2027	0.6244
Platelets (G/l)	325–888	310.5	371.5	115.093	0.7269

^a^Haematological value in BALB/c mice in Sao Paulo, Brazil – 2016

^b^*Abbreviations*: *RBC* Red blood cells, *MCV* Mean corpuscular volume, *WBC* White blood cells

## Discussion

### Microbiological state of the environment

In the present study, the average concentrations of total airborne bacteria (from 26 to 365 CFU/m^3^) and fungi (from 0 to 208 CFU/m^3^) in the animal rooms were similar to those generally found in laboratory animal houses [37]. The results of the present study confirmed that the RCI system effectively reduced numbers of both bacteria and fungi in the air compared to controls. Our previous research also demonstrated the efficacy of RCI against microorganisms in the air [38] and that the number of bacteria decreased with RCI exposure over time [25]. In the current experiment, after seven days of RCI in a conventional animal room, the number of airborne bacteria decreased by 59% (from 115 to 55 CFU/m^3^). The results of the microbiological assessment of the air by the sedimentation method were similar to those (63%) obtained by the impact method of air sampling performed under the same experimental conditions [39]. Skowron et al. obtained much better sterilization results in laboratory conditions. In their study of standardized strains of various bacteria (*Enterococcus* spp., *Clostridioides difficile*, and *Staphylococcus aureus*) in the air, the reduction ranged from 79 to 99% after 20 min of RCI treatment [40]. The diversity of the bacteria present in the air in the present study may have reduced the effectiveness of RCI. Another solution, described by Danilenko et al. [41], is a design model for a novel reactor for indoor air purification. A photocatalytic complex of ZnO and Ag material deposited on a grid support was tested in UV-purified air. Active microbial monitoring of the indoor air showed a 90% decrease in the concentration of bacteria in the room after 30–40 min. Other solutions include the use of surface photoactive pigments. The application of nano-TiO, stabilized with commercial sodium salt of polyacrylic acid (SN5040), to the paint on inside walls resulted in a nearly 100% reduction in *Escherichia coli* compared to walls painted without nano-TiO [42]. Eisenloffel et al. [43] reported that recirculating air filtration combined with UVC application reduced bacteria in a pig house by 99.4%. Airborne fungi were also spectacularly reduced (> 5 times) in the present study. While the effectiveness of photocatalysis against bacteria is well described in the literature [38, 44, 45], such a reduction in fungi in the air has not been previously reported. Generally, fungi and their spores appear to be more resilient than viruses and bacteria, being able to withstand more significant stresses caused by dehydration and rehydration, as well as UV radiation [46]. Fungal cells and spores have been shown to be more resistant to photocatalysis than bacteria, which is undoubtedly due to their cell wall properties [47]. It is known that organic material, including fungal spores, can be carried by inorganic particles (e.g. dust) [48]. It is likely that the number of fungi decreased by the seventh day of the experiment along with the reduced dust concentration in the room.

During the seven days of the experiment, the total number of bacteria in the cage bedding ranged from 47,000 to 375,000 CFU x g in the control room and from 8,000 to 163,000 CFU x g in the RCI room. The total fungal counts in the rooms were 150–13,000 CFU x g and 430–7,000 CFU x g, respectively. A study of the source of bedding contamination in laboratory rodent cages found maximal concentrations of > 6,500,000 CFU x g for bacteria and 212,000 CFU x g for fungi after four days [49].

The number of bacteria and fungi in the cage bedding remained unchanged after seven days of RCI activity. At the same time, the number of bacteria in the control cages increased by 120%, but the number of fungi decreased 13 times. The reason for these changes, independent of the photocatalysis factor, must lie in the pressure of the coexistence of the two groups of microorganisms, which has been the subject of research by many authors [50–52]. Bacteria and fungi can form a number of physical associations, whose development and functioning depend on various modes of molecular communication. Interactions between bacteria and fungi are central to many aspects of agriculture, forestry, environmental science, food production, and medicine. Bacterial-fungal interactions often cause changes in the pathogenicity or nutritional effects of one or both for plants or animals (including humans) [52]. Disinfection methods aimed at eradicating bacteria and fungi often do not take into account these interactions, while bacteria and fungi form mixed communities with virulence and immunity properties that differ significantly from those found in a single-species community [53, 54]. Pivato et al. (2009) showed that *Pseudomonas fluorescens* C7R12 can reduce wilt caused by *Fusarium*. Another study indicated that diffusible molecules from *P. aeruginosa* suppress *Aspergillus fumigatus* biofilm formation in vitro [55].

### Ammonia and dust in the air

The cages in which the animals are housed are rich in nitrogen-containing compounds that originate in urine and faeces. Microorganisms in bedding produce ammonia, one of the most irritating odorous gases formed in animal houses [55, 56]. Air samples for ammonia analysis were taken from the rooms, not from the cages, and therefore the changes in gas concentration were not spectacular. Washington and Paiton reported that over a seven-day period the ammonia concentration in rodent cages increased from about 0.7 mg/m^3^ to 17.4 mg/m^3^ [57]. In the present study, the ammonia concentration in the air of the control room increased from 1.1 mg/m^3^ to 1.3 mg/m^3^. The difference in concentration is more pronounced between the seventh day in the control room and the seventh day of the RCI experiment (1.02 mg/m^3^ and 1.3 mg/m^3^; 21%). It should be noted that at the start of the experiment the ammonia concentration was the same in both rooms. The most common way to avoid nitrogen emission into the environment is the use of absorbents as bedding additives. They combine with water and solidify bedding containing ammonia. Studies using livestock show that the addition of absorbents such as bentonite, vermiculite, or halloysite reduces the concentration of ammonia in the air by 16–90% [58–61]. Another method for reducing the release of ammonia into the environment is a well-balanced, easily digestible diet, leading to a small amount of nitrogen in the faeces. A proper diet also improves amino acid metabolism, resulting in a lower rate of both amino acid deamination and ammonia synthesis [62]. Kendall et al. reported that ammonia emissions per rat per day were reduced from 29.6 to 12.9 ppm (approximately a 56% reduction) when dietary crude protein was decreased from 12.6% to 9.35% [63].

The RCI system reduces the amount of ammonia through a photochemical process or by limiting bacterial growth. It can also be assumed that the RCI system reduced the amount of dust in the air. This is only an assumption, as in accordance with Polish regulation PN-91/Z-04030/05 only a single dust measurement was made, and thus it was not statistically significant. Air ionization results in dust sedimentation, thereby reducing transport of bacteria. Mitchell et al. used an electrostatic space charge system (ESCS) to reduce airborne dust in a small-scale broiler breeder house [64]. Ceiling fans were used to distribute negatively charged air molecules throughout the room and move negatively charged dust downwards toward the grounded bedding, where most of it was captured. The system reduced airborne dust by an average of 61%, ammonia by an average of 56%, and airborne bacteria by 67% [65]. Other researchers have reported that when an air barrier system was used in an operating room, the decrease in airborne bacteria was strongly associated with the decrease in the concentration of larger particulates (> 5 μm diameter), and airborne bacteria-carrying particles measured 4 to 20 μm. Consequently, reducing the concentration of larger airborne particulates should reduce airborne CFU density [66].

### Haematological status of mice

High concentrations of hydrogen peroxide can cause oxidative stress, inflammation, and hyperaemia in organs and tissues. In severe cases, it can cause internal haemorrhaging and air embolisms in blood vessels. Red blood cells (RBCs) are specialized and are the most abundant cells in animals and humans. All cells living under aerobic conditions are continuously exposed to ROS derived from various endogenous and exogenous sources. Exogenous sources of ROS include ionizing and non-ionizing radiation, pollutants, natural toxic gases such as ozone, drugs, and toxins, including oxidizing disinfectants. RBCs are highly susceptible to oxidative damage due to the high cell concentration of oxygen and haemoglobin, a powerful promoter of the oxidative process. They are among the first cells to be affected by adverse conditions [67]. Richards et al. [68] used multiple regression analysis to show that MCV was associated with erythrocyte distribution width and with malondialdehyde (MDA) and methaemoglobin (MetHb) levels by in chronic fatigue syndrome (CFS) patients. These changes suggest that the variation in MCV and erythrocyte morphology noted in CFS patients was associated with oxidative stress. Total leukocyte count increases significantly in response to infection, trauma, inflammation, and certain diseases [69]. Inhalation of toxic air pollutants, including exogenous reactive oxygen species, leads to cascades of signalling events that trigger the production of pro-inflammatory mediators [70]. Increased airway responsiveness, as well as augmented total white blood cell (WBC) and eosinophil counts, has been shown in animal models of asthma [71]. The results of the haematological analyses in our study only confirm the neutral influence of the RCI technology on basic health indicators. Results pertaining to lipid peroxidation in the blood, livers, lungs, and brains of mice exposed to RCI for seven days also indicated the lack of a negative oxidizing effect [39]. However, these results are not a sufficient assessment of the risk that could potentially result from hydrogen peroxide. This requires histological and immunohistochemical testing of inflammatory and oxidative stress indicators of the skin and respiratory system of animals exposed to RCI for a longer period.

## Conclusions

RCI technology applied in real time to laboratory mice reduced or inhibited an increase in bacteria and fungi and significantly decreased physical and chemical contamination within the room where the animals were housed. Although the differences obtained in the concentrations of ammonia and dust may not be of biological significance, they could be more advantageous in more contaminated environments, e.g. in poultry and pig production. While the haematology scores of the mice after RCI exposure appear promising, further investigation of more detailed animal health parameters is required. RCI could be applied in livestock buildings for other animals, to address the continually increasing threat of cross-resistance of bacteria and the lack of effective agents against drug-resistant pathogens. A decrease in ammonia concentrations in animal houses is also essential to environmental protection and may be the optimal solution for large-scale animal production buildings. Additional long-term studies are needed to confirm the safety of prolonged use of RCI systems in buildings containing animals and humans.

## Data Availability

All data supporting the conclusions of this article are included within the article.
